# Local Sleep and Alzheimer’s Disease Pathophysiology

**DOI:** 10.3389/fnins.2020.525970

**Published:** 2020-09-23

**Authors:** Bryce A. Mander

**Affiliations:** ^1^Department of Psychiatry and Human Behavior, University of California, Irvine, Irvine, CA, United States; ^2^Center for the Neurobiology of Learning and Memory, University of California, Irvine, Irvine, CA, United States

**Keywords:** Alzheimer’s disease, sleep, slow waves, sleep spindle, REM sleep, beta-amyloid protein, tau and phospho-tau protein, neurodegeneration

## Abstract

Even prior to the onset of the prodromal stages of Alzheimer’s disease (AD), a constellation of sleep disturbances are apparent. A series of epidemiological studies indicate that multiple forms of these sleep disturbances are associated with increased risk for developing mild cognitive impairment (MCI) and AD, even triggering disease onset at an earlier age. Through the combination of causal manipulation studies in humans and rodents, as well as targeted examination of sleep disturbance with respect to AD biomarkers, mechanisms linking sleep disturbance to AD are beginning to emerge. In this review, we explore recent evidence linking local deficits in brain oscillatory function during sleep with local AD pathological burden and circuit-level dysfunction and degeneration. In short, three deficits in the local expression of sleep oscillations have been identified in relation to AD pathophysiology: (1) frequency-specific frontal deficits in slow wave expression during non-rapid eye movement (NREM) sleep, (2) deficits in parietal sleep spindle expression, and (3) deficits in the quality of electroencephalographic (EEG) desynchrony characteristic of REM sleep. These deficits are noteworthy since they differ from that seen in normal aging, indicating the potential presence of an abnormal aging process. How each of these are associated with β-amyloid (Aβ) and tau pathology, as well as neurodegeneration of circuits sensitive to AD pathophysiology, are examined in the present review, with a focus on the role of dysfunction within fronto-hippocampal and subcortical sleep-wake circuits. It is hypothesized that each of these local sleep deficits arise from distinct network-specific dysfunctions driven by regionally-specific accumulation of AD pathologies, as well as their associated neurodegeneration. Overall, the evolution of these local sleep deficits offer unique windows into the circuit-specific progression of distinct AD pathophysiological processes prior to AD onset, as well as their impact on brain function. This includes the potential erosion of sleep-dependent memory mechanisms, which may contribute to memory decline in AD. This review closes with a discussion of the remaining critical knowledge gaps and implications of this work for future mechanistic studies and studies implementing sleep-based treatment interventions.

## Introduction

Both macro and micro features of sleep architecture change across the adult lifespan, and the individual variability in the magnitude of change appears to be associated with the degree of cognitive decline and the severity of risk for dementias such as Alzheimer’s disease (AD) (Ohayon et al., 2004; Redline et al., 2004; Yaffe et al., 2011; Lim et al., 2013a,b; Spira et al., 2013; Lo et al., 2014; Mander et al., 2015, 2016, 2017a; Osorio et al., 2015; Song et al., 2015; Sprecher et al., 2015, 2017; Chen et al., 2016; Bubu et al., 2017, 2019; Kabeshita et al., 2017; Pase et al., 2017; Shi et al., 2017; Tsapanou et al., 2017; Lutsey et al., 2018). While the mechanisms underlying these associations remain elusive, emerging evidence has elucidated bidirectional associations between biological mechanisms of aging and AD and the expression of sleep processes related to neuroplasticity (Ju et al., 2014; Mander et al., 2016). In particular, certain micro features of sleep are expressed locally within specific brain circuits supporting processes regulating systemic and synaptic neuroplasticity (McGaugh, 2000) in order to facilitate memory formation and consolidation (Buzsaki, 1998; Steriade, 2006; Walker, 2009; Diekelmann and Born, 2010; Wamsley and Stickgold, 2011; Watson and Buzsaki, 2015). New findings indicate that topographic and frequency-specific disruptions in the expression of these brain oscillations, including slow waves and sleep spindles during non-rapid eye movement (NREM) sleep and EEG desynchrony during REM sleep, occur in the context of AD pathophysiology—in some cases even prior to onset of mild cognitive impairment (MCI)—and these local sleep deficits differ from those observed in normal aging (Hassainia et al., 1997; Petit et al., 2004; Jyoti et al., 2015; Mander et al., 2015, 2016, 2017a; Brayet et al., 2016; Gorgoni et al., 2016; De Gennaro et al., 2017; Holth et al., 2017; Lucey et al., 2019). It is therefore possible that certain local sleep deficits may reflect an abnormal aging process in its initial stages, and that disruptions in sleep-dependent memory mechanisms may contribute to the biological and cognitive consequences of AD pathophysiology. In this review, we summarize the state of the science linking local sleep to core features of AD pathophysiology and AD-related hippocampus-dependent memory impairment.

## Local Sleep

The brain expresses a complex and dynamic organization of oscillatory activities during sleep, cycling between NREM and REM sleep stages that each distinctly organize local brain activities across frequency, topography, and time (Landolt et al., 1996; Liscombe et al., 2002; Steriade, 2006; Diekelmann and Born, 2010; Staresina et al., 2015; Watson and Buzsaki, 2015; Sprecher et al., 2016; Helfrich et al., 2018; Scarpelli et al., 2019). The spatially circumscribed nature of the expression of these sleep-specific oscillatory activities and their frequency specificity highlight the local nature of some canonical sleep features. For example, most NREM slow wave and sleep spindle oscillations are expressed regionally and migrate across cortex in a predictable manner (Massimini et al., 2004; Nir et al., 2011; Muller et al., 2016). Further, in cases of excessive sleep pressure, isolated neuronal ensembles have even been shown to express these sleep-specific rhythms while the majority of cortex remains in a state of wakefulness (Van Dongen et al., 2011; Krueger et al., 2019). The fact that this occurs has led to the hypothesis that sleep is not entirely global, i.e., it does not always occur equally throughout the entire brain (Van Dongen et al., 2011; Krueger et al., 2019). Furthermore, even during sleep local expression of certain oscillatory activities, such as slow waves during NREM sleep, can be influenced by prior waking experiences in a topographically-specific manner. More specifically, neuronal ensembles more active during learning will exhibit greater local intensity of slow wave activity relative to surrounding cortex (Huber et al., 2004, 2006). These phenomena demonstrate that certain sleep features are local and not global.

Two specific NREM sleep oscillations of note that are organized in this fashion are slow waves and sleep spindles. Slow waves, defined as low frequency (0.5–4.5 Hz) and high amplitude (>75 μV) waveforms, are generated in cortex, predominantly within association cortices including medial prefrontal cortex, insular cortex, and the cingulate cortex (Steriade, 2006; Murphy et al., 2009). Their expression is dependent on the coordinated synchrony of hyperpolarized down states and depolarized up states of local neuronal ensembles (Steriade et al., 1993b; Steriade, 2006). The spectral power of slow waves, termed slow wave activity (SWA), peaks over frontal electroencephalography (EEG) derivations in the first quartile of the night, dissipating across the sleep period in a manner reflective of a homeostatic “Process S” (Borbely et al., 1981; Borbely, 1982; Landolt et al., 1996; Carrier et al., 2001; Mander, 2013; Mander et al., 2017a). Recent optogenetic findings support the existence of two kinds of slow waves: lower frequency slow oscillations (SO; <1 Hz) that support memory consolidation processes and faster delta waves (1–4.5 Hz) that facilitate forgetting (Steriade, 2006; Kim et al., 2019). Whether the temporal or topographical organization of these waveforms is distinct or is influenced by aging or AD differentially remains to be seen, and should be a focus of future studies.

A second characteristic sleep oscillation that is critically tied to neuroplasticity is the sleep spindle. Sleep spindles are transient (0.5–3 s) bursts of oscillatory activity in the sigma frequency range (11–16 Hz) with waxing and waning components. They are generated within the reticular nucleus of the thalamus and expressed through cortico-thalamic loops (De Gennaro and Ferrara, 2003; Steriade, 2006). Two kinds of sleep spindles have been described as well: fast (∼13–16 Hz) and slow (∼11–13 Hz) frequency sleep spindles (Jobert et al., 1992; De Gennaro and Ferrara, 2003). Though exact frequency definitions vary by study, the distinct topographic nature of their expression does not. Fast frequency sleep spindles tend to peak over midline central and parietal EEG derivations, while slow frequency sleep spindles tend to peak in expression over frontal EEG derivations (Jobert et al., 1992; De Gennaro and Ferrara, 2003). Moreover, while SWA may dissipate across the sleep period, spindle activity tends to peak in the morning (Landolt et al., 1996). However, these NREM sleep oscillations are not expressed in isolation, tending to couple during the rising phase of the depolarizing slow wave up state (Steriade et al., 1993a; Steriade, 2006; Staresina et al., 2015). This phase-locking is specific to the SO and is also accompanied by phase locking of another oscillation, the hippocampus ripple, within the troughs of the sleep spindle oscillation (Staresina et al., 2015; Helfrich et al., 2018). Coordinated firing of neuronal ensembles during initial encoding has been observed to spontaneously reoccur during ripples (Wilson and McNaughton, 1994; Ji and Wilson, 2007; Davidson et al., 2009), with ripples instigating information transfer from the hippocampus to the cortex when phase locked to SOs and sleep spindles (Olcese et al., 2018; Helfrich et al., 2019). The coupling of these three cortical, thalamic, and hippocampal brain oscillations, and the “replay” associated with them, is thought to support the consolidation of memories acquired during prior wakefulness (Steriade, 2006; Diekelmann and Born, 2010; Watson and Buzsaki, 2015; Helfrich et al., 2018, 2019). Indeed, recent evidence demonstrates that stimulation and suppression of sleep spindles only impacts sleep-dependent memory consolidation if it occurs during the rising phase of the depolarizing up-state and in coordination with hippocampal ripples (Latchoumane et al., 2017).

The spectral content and topographic organization of REM sleep differs from that observed in NREM sleep. The apparent wake-like nature of EEG-measured neuronal activity during REM sleep is why it is also known as “paradoxical sleep” (Peever and Fuller, 2016), though there are important distinctions that are observed when using other neuroimaging modalities (Braun et al., 1997; Maquet, 2000) or analytic approaches (Lendner et al., 2020). Of particular note, while the low voltage mixed frequency content of the “desynchronized” EEG observed during wakefulness is supported by a series of brainstem, midbrain, and hypothalamic glutamatergic, monaminergic, and cholinergic inputs, cholinergic input plays a more dominant role in generating the desynchonized EEG in REM sleep than monaminergic inputs (Peever and Fuller, 2016; Scammell et al., 2017). Thus, while the EEG looks the same in wake and REM sleep, the neurobiological correlates are distinct.

In terms of spectral content, both alpha and theta power are particularly prominent in REM sleep (Landolt et al., 1996), with theta peaking over fronto-central derivations, and alpha peaking over occipito-parietal derivations (Scarpelli et al., 2019), Similar to theta activity, higher frequency content, including beta and gamma power, peak over fronto-central derivations, but while low frequency power, including delta and theta power decrease across successive REM sleep periods, high frequency content remains relatively stable (Liscombe et al., 2002). Little is known about the functional relevance of the organized expression of EEG activities during REM sleep, and this should be a topic of future study.

Substantial evidence indicates that both aging and AD influence local NREM and REM sleep expression in distinct ways, and these effects are reviewed below.

## Local Sleep in Aging and Alzheimer’s Disease (AD)

### Aging

Increasing age is associated with reductions in oscillatory activities across multiple frequency bands in non-rapid eye movement (NREM) sleep (Sprecher et al., 2016). The biggest reductions occur globally across the frequency bands of slow waves (0.5–4.5 Hz) and sleep spindles (12–16 Hz), with the largest reductions occurring over frontal EEG derivations, due to the reduced incidence and amplitude of slow wave and sleep spindle oscillations (Dijk et al., 1989; Landolt et al., 1996; Carrier et al., 2001; Mander, 2013; Martin et al., 2013; Mander et al., 2014, 2017a,b; Sprecher et al., 2016). Aging also disrupts the phase-locked synchrony between slow waves and sleep spindles, with slow wave-sleep spindle coupling being more variable and occurring at an earlier phase of the slow wave, closer to the hyperpolarized slow wave down state (Helfrich et al., 2018; Muehlroth et al., 2019). All these age effects on NREM sleep oscillation expression have been associated with age effects on frontal and hippocampal gray and white matter volume and integrity (see Mander et al., 2017a, for an in depth review). Central to this work is the consideration of whether these age-related changes contribute to cognitive decline in aging, and this will be reviewed in detail later in this review.

In addition to NREM sleep, a few studies have explored age effects on REM sleep architecture. These studies have shown that older adults experience a modest reduction in REM sleep duration (0.6% per decade) that emerges much later than age effects on NREM sleep (Ehlers and Kupfer, 1997; Van Cauter et al., 2000; Carrier et al., 2001; Gori et al., 2004; Ohayon et al., 2004; Redline et al., 2004; Floyd et al., 2007; Scarpelli et al., 2019). However, qualitative differences in REM sleep are also present in older adults even if REM duration reductions are minimal, including increased awakenings from REM sleep, decreased REM latency, decreased REM density, and shorter and more disorganized REM bursts, particularly in adults over 65 years (Gillin et al., 1981; Ehlers and Kupfer, 1989, 1997; Vegni et al., 2001; Darchia et al., 2003; Conte et al., 2014). In terms of REM sleep microarchitecture, a few reports have indicated spectral power is reduced across delta, theta, and alpha frequencies in older adults, particularly over central derivations (Landolt et al., 1996; Scarpelli et al., 2019). It remains unknown why REM sleep microarchitecture is reduced in aging, and whether these changes are functionally relevant or epiphenomenal.

### Alzheimer’s Disease

**FIGURE 1:**
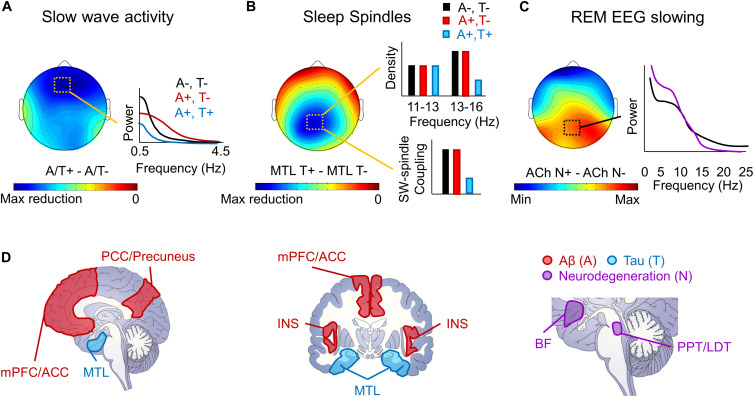
Schematic depiction of local sleep features disrupted by AD pathophysiology. **(A)** A schematic topoplot of decreases in SWA during NREM sleep in individuals with Aβ (A +, red) and tau (T +, blue) pathology relative to those without pathology (A–, T–, black) is presented, with cooler colors reflecting the severity in the hypothesized reduction in SWA. A schematic of the effects of Aβ and tau pathology on SWA by frequency are plotted to the right. **(B)** A topoplot of decreases in sleep spindles in individuals with tau pathology, particularly in the medial temporal lobe (MTL) is presented, with cooler colors reflecting the severity of sleep spindle loss in individuals with tau pathology. Schematic bar graphs of the effects of both Aβ (red) and tau (blue) pathology on sleep spindle density and slow wave (SW)-sleep spindle coupling relative to those without AD pathology (black) are presented to the right. **(C)** A topoplot of the increase in REM sleep EEG slowing in those with cholinergic degeneration (ACh N +, purple) relative to those without significant cholinergic degeneration (ACh N–, black). A schematic frequency by spectral power plot of REM sleep is shown to the right, indicating increased low frequency power and decreased high frequency power in the presence of cholinergic degeneration. **(D)** Schematic of brain slices depicting the hypothesized locations of Aβ (A, red) and tau (T, blue) deposition, as well as neurodegeneration (N, purple), that lead to the observed local deficits in NREM and REM sleep. mPFC/ACC denotes medial prefrontal cortex and anterior cingulate cortex, PCC/Precuneus denotes posterior cingulate cortex and precuneus, MTL denotes the medial temporal lobe, INS denotes the insula cortex, BF denotes the cholinergic basal forebrain, and PPT/LDT denotes the cholinergic pedunculopontine and lateral dorsal tegmental nuclei.

Local sleep deficits have been observed in the context of MCI, AD, and even in healthy older adults with AD pathology (Figure 1). These local sleep deficits diverge from those observed in the context of normal aging both in terms of topography and frequency (Petit et al., 2004; Mander et al., 2016, 2017a). Reports have shown that slow wave sleep (SWS) is reduced and more fragmented in MCI, AD, and in rodent models of AD (Prinz et al., 1982; Gagnon et al., 2008; Hita-Yanez et al., 2012, 2013; Roh et al., 2012; Westerberg et al., 2012; Mander, 2013; Jyoti et al., 2015; Kam et al., 2016), SWA is lower over midline frontal, central, and parietal derivations in healthy older adults with AD pathology and patients with MCI (Westerberg et al., 2012; Mander et al., 2015; Varga et al., 2016b; Lucey et al., 2019), and there are fewer frontal K-complexes in AD (De Gennaro et al., 2017). However, this effect of AD pathology and diagnosis on slow wave expression appears to depend on slow wave frequency, particular AD pathology, and, potentially, disease stage (Figure 1A). More specifically, in healthy older adults with cortical Aβ pathology, SWA deficits appear to be specific to lower frequencies (i.e., in the SO frequency range; < 1 Hz), with increases in SWA observed in the delta frequency range (1–4 Hz) (Mander et al., 2015; Kastanenka et al., 2017, 2019). This effect may change to reflect a more global loss of SWA across frequencies once tau pathology reaches the cortex in the MCI stage (De Gennaro et al., 2017; Lucey et al., 2019). Similar findings were observed in transgenic mouse models of AD, though this depended on which model was examined. Decreases in fronto-parietal SWA and increases in higher frequency power were observed during NREM sleep in Tg2576 and APP/PS1 transgenic mouse models of AD, but not in 3xTgAD mice (Zhang et al., 2005; Kent et al., 2018). This is noteworthy given that Tg2576 and APP/PS1 transgenic AD mouse models both exhibit rapid increases in Aβ pathology in the absence of neurofibrillary tangles, while the 3xTgAD mice express a more mild level of both Aβ and tau pathology. Together, these findings indicate that the influence of AD pathophysiology on SWA may not be fixed, but instead may change depending on the location and degree of burden of distinct AD pathologies.

Reduction in sleep spindle expression has also been observed in the context of AD. There are fewer sleep spindles and lower spindle activity in patients with MCI and AD (Rauchs et al., 2008; Westerberg et al., 2012; Gorgoni et al., 2016). However, this effect appears to be specific to faster frequencies (13–16 Hz) over parietal derivations (Figure 1B), with no discernable effect on frontal sleep spindles in slower frequencies (11–13 Hz) (Westerberg et al., 2012; Gorgoni et al., 2016). This is in stark contrast to the effect of age, which selectively disrupts frontal sleep spindles regardless of frequency, leaving parietal fast frequency sleep spindles largely spared (Martin et al., 2013; Sprecher et al., 2016; Mander et al., 2017a). The reduction of sleep spindles, and in particular fast frequency sleep spindles, is also apparent in healthy older adults with tau pathology (Kam et al., 2019). The effect of AD pathologies and MCI and AD diagnosis on slow wave-sleep spindle coupling remain unclear, though a recent study showed that tau pathological burden in the medial temporal lobe was associated with reduced slow wave-sleep spindle coupling (Winer et al., 2019). Because these parietal fast frequency sleep spindle deficits associated with MCI and AD are so topographically distinct from age-related deficits in sleep spindles, it is possible that these deficits could represent an early physiological signal distinguishing between abnormal and normal aging processes.

A robust literature demonstrates that MCI and AD diagnosis is associated with characteristic changes in REM sleep microarchitecture in a topographically-specific manner (Figure 1C). Specifically, a loss of EEG desynchrony during REM sleep is observed as early as MCI (Brazete et al., 2013; Brayet et al., 2016), and can be used to differentiate individuals with and without AD with high diagnostic accuracy (Hassainia et al., 1997). This measure, quantified as the ratio of low frequency to high frequency power can even distinguish between individuals with amnestic versus non-amnestic MCI (Brayet et al., 2016), but can also predict non-AD dementia onset and cognitive decline in individuals with REM behavior disorder (Brazete et al., 2016). It is therefore possible that this signature may be more specific to dementia-related cognitive decline than AD, *per se*. The effect of MCI and AD diagnoses on EEG desynchrony is particularly prominent over central and posterior EEG derivations (Hassainia et al., 1997; Brazete et al., 2013, 2016; Brayet et al., 2016). Alongside these findings, others have found loss of central-parietal theta activity during REM sleep in patients with amnestic MCI (Westerberg et al., 2012), and both AD and tauopathy rodent models show reduced theta and alpha activity (Holth et al., 2017). Hence, the quantitative expression of REM sleep EEG is also impacted by MCI and AD, and these deficits may track cognitive decline associated with dementia.

The distinct nature of the effects of AD pathophysiology on the expression of local sleep features relative to those observed with advancing age hints at a distinction in underlying mechanisms, which may herald the emergence and progression of AD in its initial stages. Recent work highlights the mechanistic links between local sleep disturbance and AD pathophysiological features, which are examined in detail in the next section.

## Emerging Links Between Local Sleep and AD Pathophysiology

### The Alzheimer’s Disease Cascade—The Role of Amyloid (A), Tau (T), and Neurodegeneration (N)

**FIGURE 2:**
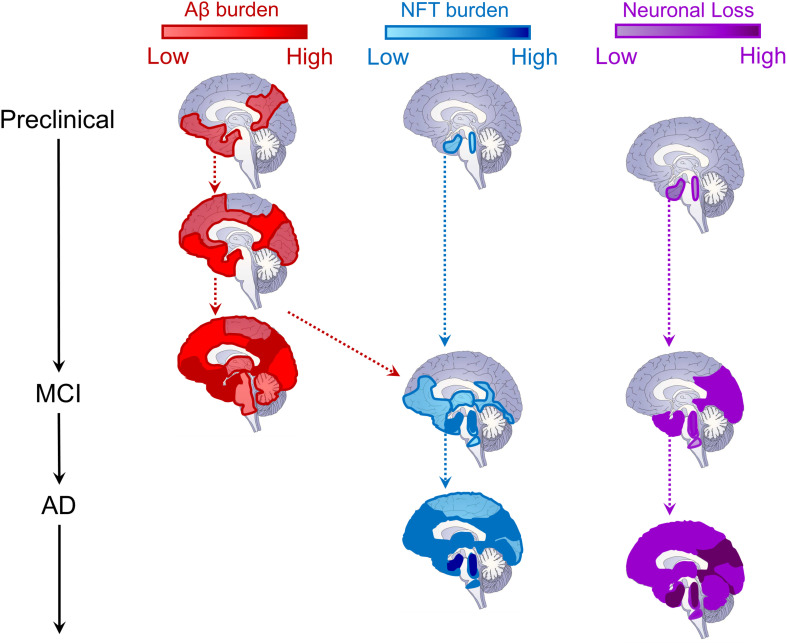
Schematic depiction of the evolution of Alzheimer’s disease biomarkers. Sagittal brain slices depicting the hypothesized locations of Aβ (red, left column) and tau (blue, middle column) deposition, as well as neurodegeneration (neuronal loss, purple, right column) across disease stages. For Aβ, deposition begins in medial prefrontal, cingulate, temporal, and precuneus regions, and progresses cortically and then subcortically until most of the brain contains amyloid plaques (Braak and Braak, 1991; Thal et al., 2002; Sepulcre et al., 2013; Palmqvist et al., 2017). The cortex is likely already saturated with amyloid plaques by the time most patients convert to MCI (Jack et al., 2010, 2013). For tau neurofibrillary tangles (NFT), deposition begins in the locus coeruleus and cholinergic brainstem nuclei in Braak stage 0, progressing into entorhinal cortex in the medial temporal lobe by stage I (Braak and Braak, 1991; Mesulam et al., 2004; Grudzien et al., 2007; Ehrenberg et al., 2017). NFTs remains largely constrained within the MTL until Braak stage III (Braak and Braak, 1991, 1995), when, through its interaction with Aβ pathology (red dashed arrow), tau pathology spreads throughout the brainstem, thalamus, MTL, inferior temporal cortex, and medial prefrontal and cingulate brain regions (Vogel et al., 2020). It is at this time that MCI conversion begins to be observed (Jack et al., 2010, 2013). In later Braak stages, tau pathology is observed throughout most of the cortex (Braak and Braak, 1991, 1995), triggering widespread cortical degeneration as its spreads (Jack et al., 2010, 2013). Regarding neurodegeneration (neuronal loss), as with NFTs, synaptic and neuronal loss begins within locus coeruleus, cholinergic brainstem nuclei, and the MTL (Mesulam et al., 2004; Grudzien et al., 2007; Whitwell, 2010). As MCI progresses into later stages, cortical atrophy is prevalent throughout the temporal, parietal, and occipital lobes (Whitwell, 2010). Ultimately, in later AD stages, cortical atrophy progresses through lateral frontal cortex and, eventually, throughout the entire brain, impacting neural regulation of even basic functions supporting life (Jack et al., 2010, 2013; Whitwell, 2010).

Contemporary theories of AD pathogenesis describe the emergence of a biological cascade of pathological events which trigger a progressive degenerative process that ultimately results in the clinical and cognitive symptoms of dementia (Jack et al., 2010, 2013; Jagust, 2018; Marquez and Yassa, 2019). While there remains controversy over which pathological event emerges first (see Herrup, 2015; Musiek and Holtzman, 2015, for further details), what is clear is that the initial stages involve the build-up of cortical Aβ (A) and subcortical tau (T; Figure 2). As they converge and interact, tau begins to spread cortically and Aβ subcortically, triggering progressive and widespread neurodegeneration (N) which results in progressive cognitive and clinical dysfunction, and ultimately gross loss of basic functions (Jack et al., 2010, 2013, 2019).

The probability of onset and rate of progression of this neurodegenerative process is influenced by other biological factors which directly or indirectly influence this core biology of AD. For example, many of the known genetic risk factors for familial and sporadic AD, such as mutations in the amyloid precursor protein (APP), presinilin, and apolipoprotien E (APOE) genes, result in an acceleration of Aβ accumulation and the resulting neurodegeneration (Bagyinszky et al., 2014). Sex also influences AD, with women being at twofold greater risk for developing AD relative to men (Podcasy and Epperson, 2016). Recent evidence indicates that one potential reason for this is that the same degree of Aβ burden may facilitate a greater degree of accumulation of tau pathology in women relative to men (Buckley et al., 2019).

Emerging evidence indicates that chronic inflammation plays a critical role in mediating the interactions between multiple pathophysiologic features of AD, facilitating its progression (for in depth reviews see, Heppner et al., 2015; Spangenberg and Green, 2017; Wang et al., 2017). Microglia—glial cells supporting innate immunity within the central nervous system—are activated by Aβ through its interaction with the triggering receptor expressed on myeloid cells 2 (TREM2), resulting in the release of proinflammatory cytokines and chemokines, including interleukin-1 β (IL-1β), IL-6, tumor necrosis factor alpha (TNF-α), among others (Heppner et al., 2015; Spangenberg and Green, 2017). This activation, alongside activation of astrocytes, facilitates Aβ clearance through degradation and phagocytosis (Heppner et al., 2015; Spangenberg and Green, 2017). When Aβ plaques are formed, both astrocytes and microglia surround them to degrade and clear them from the central nervous system, as well as to form a barrier to minimize the toxic effects of Aβ on surrounding neural tissue (Heppner et al., 2015; Spangenberg and Green, 2017).

Unfortunately, Aβ also impairs microglial function, suppressing its ability to degrade and clear Aβ. This leads to the induction of a chronic inflammatory state, which facilitates microglial burn-out, astrocytic atrophy, sustained Aβ production, decreased Aβ clearance, disrupted tau-microtubule binding, neurofibrillary tangle (NFT) formation, and propagation of tau pathology (Heppner et al., 2015; Spangenberg and Green, 2017). Chronically activated microglia also contribute to neurodegeneration and cognitive decline through dysfunctional phagocytosis, which results in pathological stripping of synapses and facilitation of neuronal loss (Spangenberg et al., 2016), a process also observed following sleep deprivation (Bellesi et al., 2017). Indeed, the number of activated microglia have been shown to correlate with cognitive decline (Cagnin et al., 2001; Versijpt et al., 2003; Edison et al., 2008). These findings are supported by studies in humans showing that chronic systemic inflammatory conditions, such as rheumatoid arthritis, increase risk for developing AD through sustained increases in IL-1β, IL-6, TNF-α, and C-reactive protein (CRP) levels, which can cross the blood brain barrier and directly impact brain Aβ metabolism or impact Aβ in the periphery which then interacts with central Aβ pools (Wang et al., 2017). Further, in addition to genetic mutations in genes impacting Aβ accumulation, genetic mutations in numerous genes affecting microglial function, such as mutations in TREM2, also increase AD risk (Spangenberg and Green, 2017). In addition, release of APOE by astrocytes is thought to critically support the capacity of microglia to clear Aβ (Terwel et al., 2011), and thus mutations in the APOE gene may impact Aβ in part through its impact on central immunity. It is important to note, however, that chronic inflammation can precede Aβ plaque deposition, and facilitate AD pathogenesis (Krstic et al., 2012), indicating that chronic inflammation may not only be an outcome of Aβ pathophysiology but also an initiating event. Thus, onset into and progression of AD depends on the interaction between two distinct pathologies, that of Aβ and tau, and the influence of other biological factors that affect them, their interaction, and potentially their impact on neuronal integrity and function.

Prior to widespread degeneration, Aβ and tau also influence neuronal function in distinct ways. A recent study implemented multiple rodent models and examined neuronal dysfunction resulting from them (Busche et al., 2019). In a model expressing only Aβ, neuronal hyperexcitability was predominant (Busche et al., 2019), mirroring the dysfunctional hippocampus and default mode network hyperactivation observed in early stages of MCI and Aβ positive but cognitively asymptomatic older adults (Sperling et al., 2009; Yassa et al., 2010b; Leal et al., 2017). This is also consistent with other studies reporting increased incidence of interictal spikes, particularly during sleep, in rodent models overexpressing Aβ pathology (Kam et al., 2016). In further support of this notion, recent studies have shown that antiepileptic drugs targeting this presumably Aβ-related hyperactivity resolved hippocampus-dependent memory impairments observed in early stages of amnestic MCI (Bakker et al., 2012, 2015). In contrast, rodent models expressing either tau alone or both Aβ and tau resulted in neuronal silence (Busche et al., 2019), meaning that, when convergent, tau trumps the effects of Aβ on neuronal function. This finding supports the possibility that the form of neuronal dysfunction observed in AD, and the cognitive consequences of that dysfunction, may depend on the neural network examined and the relative abundance of both Aβ and tau pathology within that network. This theoretical model of AD offers critical insight into the mechanistic links between local NREM and REM sleep dysfunctions and AD (Figure 1D). It also highlights the potential of these local sleep dysfunctions to offer a window into AD stage and progression, and the cognitive consequences of their relationships, all of which are reviewed below.

### β-Amyloid and Local Expression of Slow Wave Activity (A)

Aβ levels increase during wakefulness and decrease during sleep in the interstitial fluid (ISF) in rodents and CSF in humans, and subjective and objective measures of sleep duration and quality are associated with Aβ burden (Kang et al., 2009; Roh et al., 2012; Ju et al., 2013; Spira et al., 2013; Mander et al., 2015; Sprecher et al., 2015, 2017; Varga et al., 2016b), indicating that Aβ peptides are regulated by the sleep/wake cycle. Collectively, these studies show that NREM sleep loss is associated with greater Aβ burden while greater Aβ burden further disrupts NREM sleep, potentially triggering a vicious cycle fostering AD progression (Ju et al., 2014; Mander et al., 2016). The mechanisms for these bidirectional relationships remain unclear, though there are a few candidates highlighted by recent work. First and foremost considers how NREM sleep disruption may result in increased Aβ burden. Aβ burden increases with increasing neuronal activity (Li et al., 2013), and neurometabolic activity is higher in wake than in SWS (Buchsbaum et al., 1989; Braun et al., 1997; Dworak et al., 2010). Thus, reductions in SWS duration increases the duration of wakefulness which then results in increased net Aβ accumulation.

A second possible mechanism regards findings linking disruptions in slow oscillation expression in APP transgenic mice to Aβ plaque burden (Kastanenka et al., 2017, 2019). In APP transgenic mice, SWA is reduced specifically in the slow oscillation frequency range (<1 Hz) by 3 months of age, 2 months before Aβ plaques are apparent (Kastanenka et al., 2017). This is accompanied by reduced GABA levels and downregulation of GABA_A_ and GABA_B_ receptor expression, which directly impacted the expression of slow oscillations by disrupting neuronal synchrony. Slow oscillation expression was restored by both increasing GABA levels and by optogenetically enhancing neuronal synchrony, and this resulted in reduced Aβ plaque burden and intracellular calcium overload (Kastanenka et al., 2017). In contrast, doubling the frequency of the slow oscillation to >1 Hz resulted in increased Aβ production, increased intracellular calcium overload, and decreased dendritic spine density (Kastanenka et al., 2019).

Yet another possibility involves the recently discovered glymphatic system, which is a biological process in the brain that actively clears toxins and waste from the ISF by flushing the brain with CSF pulses (Jessen et al., 2015; Rasmussen et al., 2018). Two studies actively link this process to SWS. First, glymphatic flow is increased during SWS and results in increased clearance of Aβ proteins in rodents (Xie et al., 2013). Second, SWA peaks prior to and is phase locked to CSF flow through the brain in humans (Fultz et al., 2019). These findings suggest that it may not be NREM sleep, *per se*, but the expression of slow waves that is mechanistically linked to glymphatic clearance during sleep. If true, the efficacy of this system could depend on the integrity of astrocytic mechanisms regulating neuronal function. Astrocytes directly regulate the transition to and expression of slow oscillations, potentially through spatial K^+^ buffering through the inwardly rectifying potassium channel Kir4.1 expressed in astrocytes in tripartite synapses (Fellin et al., 2009; Poskanzer and Yuste, 2016; Haydon, 2017; Cucchiara et al., 2020). Astrocytic function is also central to the expression of the glymphatic system (Nedergaard, 2013). However, high Aβ burden in AD results in loss of Kir4.1 potassium channels, as well as aquaporin 4 (AQP4) channels which are also hypothesized to be critical for glymphatic function (Wilcock et al., 2009; Nedergaard, 2013; Kress et al., 2014; Zeppenfeld et al., 2017). Hence, disruptions in slow waves during NREM sleep, associated with astrocytic dysfunction, may then decrease active clearance of AD pathology, also potentially yielding a net increase in Aβ accumulation. These possibilities are supported by recent studies showing significant associations between slow wave incidence and power and Aβ burden (Zhang et al., 2005; Mander et al., 2015; Varga et al., 2016b; Kastanenka et al., 2017; Kent et al., 2018). Directly addressing this possibility, a recent study showed that active suppression of SWA using acoustic stimulation resulted in increased CSF Aβ levels in middle-aged adults, indicating the potential for a causal relationship (Ju et al., 2017). However, it is important to note that the role of glymphatic clearance during SWS on Aβ protein levels has only been shown using injected radiolabeled Aβ proteins, and no study to date has shown direct evidence for endogenous clearance of Aβ via the glymphatic system during SWS (Smith and Verkman, 2018). This limitation is a critical caveat that must be addressed in future studies.

Lastly, it has been hypothesized that another potential pathway linking sleep deficits to increased Aβ pathology and AD risk is through an association with chronic inflammation (Irwin and Vitiello, 2019). Aging is coincidentally associated with chronic inflammation, SWS disruption, and increased AD risk (Ingiosi et al., 2013; Besedovsky et al., 2019). Sleep, and in particular SWS, has a complex bidirectional interaction with biological processes supporting immune function (for in depth reviews see, Besedovsky et al., 2019; Irwin, 2019). A recent meta-analysis of over 70 studies indicated that evidence of sleep disturbance, short sleep duration, and excessively long sleep duration was associated with elevated plasma levels of CRP and IL-6 (Irwin et al., 2016). Further, short sleep duration, greater wake after sleep onset (WASO), worse sleep efficiency, and greater of percentages of the sleep period with blood oxygen saturations below 90% are associated with increased IL-6, CRP, TNF-α, and interferon γ (INF- γ) in older adults and AD caregivers (Friedman et al., 2005; Von Kanel et al., 2006; Smagula et al., 2016). In addition, experimental sleep restriction to four hours increased nuclear factor κ-light-chain-enhancer of activated B cells (NF- κB) in plasma, which is a critical factor regulating the inflammatory signal cascade (Irwin et al., 2008). Moreover, chronic sleep restriction to 4 h per night for 12 days resulted in increased IL-6 plasma levels in young adults (Haack et al., 2007).

In addition to sleep disturbance triggering an inflammatory response, inflammation directly impacts sleep expression as well. However, the specifics of this effect depend on the duration and magnitude of inflammatory activation. While acute inflammation can induce SWS and enhance SWA, chronic inflammatory conditions are associated with fragmented sleep, alpha intrusions in NREM sleep, and reduced SWS, SWA, and REM sleep (Besedovsky et al., 2019; Irwin, 2019). Together these findings portray a complex interaction between inflammation and SWS in later life, with age-related increases in chronic inflammation potentially impairing SWS expression and overall sleep quality, ultimately resulting in greater systemic inflammation. Since chronic inflammation promotes Aβ accumulation (Krstic et al., 2012; Heppner et al., 2015; Spangenberg and Green, 2017), SWS deficits may increase Aβ burden through an interaction with markers of systemic and central inflammation. However, it should be noted that data linking either systemic or central inflammation markers to the local expression of any sleep oscillation is limited. Future work should examine this more closely to more fully address the hypothesis that slow wave deficits increase Aβ burden through interactions with inflammatory processes.

The second mechanistic question involves how Aβ burden may impact the expression of slow waves. NREM sleep is disrupted in rodent models over-expressing Aβ (Roh et al., 2012). Moreover, our recent work has shown that there is an association between SWA and cortical Aβ burden in cognitively asymptomatic older adults, but that this association is distinct from that due to age and age-related frontal cortical atrophy (Mander et al., 2015). Aβ was not associated with a global loss of slow waves or a global reduction in SWA, as is common in aging (Mander et al., 2017a), but instead showed a negative association with slow waves and SWA in the SO frequency range (0.6–1 Hz) and a positive association with slow waves and SWA in the delta frequency range (1–4 Hz), representing a shift in the slow wave distribution (Figure 1A) (Mander et al., 2015; Winer et al., 2019). If Aβ did not disrupt slow waves, but only was increased by slow wave disruption, than it would be expected that age-related changes in slow waves would predict increases in Aβ burden. This was not what we found. Instead, deficits associated with SWA were specific to Aβ and independent of age, indicating that Aβ may further disrupt slow wave expression independently of age-related changes. Interestingly, a similar effect on the SWA distribution was also observed in APP transgenic mice, a rodent model of AD selectively overexpressing Aβ pathology, with the loss of low frequency SO activity emerging as early is 3 months of age (Kastanenka et al., 2017). The mechanisms for how Aβ may disrupt slow wave expression is unknown, though there are some theoretical possibilities. One possibility is that Aβ may disrupt the generation and expression of slow waves as it accumulates in cortical slow wave generators (Figures 1A,D). Indeed, Aβ plaques initially deposit preferentially within many slow wave generating regions, including within the medial prefrontal cortex (mPFC) and anterior and posterior cingulate gyri (Buckner et al., 2005; Murphy et al., 2009; Sepulcre et al., 2013; Jagust, 2018). Supporting this possibility, the association between Aβ burden and SWA is not global, but peaks locally over midline and frontal derivations (Mander et al., 2015; Varga et al., 2016b). Further, source analysis confirmed that the strongest predictor of frontal SWA was Aβ burden within the same mPFC region where frontal slow waves were sourced (Mander et al., 2015). It remains to be determined exactly how Aβ burden could disrupt slow wave generation and expression. One hypothesis is that Aβ directly acts on the neurobiological mechanisms controlling slow wave expression (Mander et al., 2016). There is some evidence that Aβ disrupts GABA levels and GABA and NMDA receptor signaling (Kurup et al., 2010; Busche et al., 2015; Kastanenka et al., 2017), which are critical for SO expression (Steriade et al., 1993b). Another potential mechanism is the effect of Aβ on the inwardly rectifying potassium channel Kir4.1 in astrocytes (Wilcock et al., 2009). Astrocytes have been shown to impact slow oscillation expression through an impact on NMDA receptor activity in neurons (Fellin et al., 2009). Further, a recent report has linked a gain of function mutation in Kir4.1 channels in children expressing the Autism-Epilepsy Phenotype with a lengthening of the slow oscillation period during NREM sleep (Cucchiara et al., 2020). Since the presence of Aβ results in a loss of Kir4.1 channels (Wilcock et al., 2009), rather than a gain of function, it is likely that Aβ could trigger the opposite effect, i.e., a shortening of period and thus an increase in the mean slow oscillation frequency. This hypothesis remains to be tested and should be a focus of future studies. Yet another possibility is that Aβ burden triggers generalized cortical hyperexcitability, which then results in a shift toward a greater abundance of delta waves and a reduction in SO events as a consequence. Indeed, optogenetically doubling the slow oscillation frequency from 0.6 to 1.2 Hz in APP transgenic mice resulted in increased Aβ production, supporting the frequency specificity and bidirectional nature of this effect (Kastanenka et al., 2019). Lastly, since Aβ increases markers of inflammation and chronic inflammation can disrupt SWA expression, inflammation may also contribute to deficits in SWA associated with Aβ (Besedovsky et al., 2019; Irwin, 2019). Supporting the frequency specificity of this effect, relative to healthy controls, patients with myalgic encephalomyelitis/chronic fatigue syndrome (ME/CFS) exhibit reduced SWA in the SO frequency range and increased SWA in the delta frequency range, particularly over prefrontal cortex (Le Bon et al., 2012). This is notable, because central inflammation is a core component of ME/CFS (VanElzakker et al., 2019) and this frequency effect on SWA is highly consistent with the reported effects of Aβ (Mander et al., 2015; Winer et al., 2019). Regardless of the mechanism or the direction of causality, what is clear is that Aβ burden is associated with local disruptions in frontal slow waves, even prior to MCI diagnosis both cross-sectionally (Mander et al., 2015; Varga et al., 2016b; Winer et al., 2019) and longitudinally (Winer et al., 2020 in press). Some evidence also indicates that this effect of Aβ burden on local sleep may be specific to slow wave expression, as it does not appear to be associated with the REM sleep and spindle deficits observed in MCI and AD (Brazete et al., 2013, 2016; Mander et al., 2015; Brayet et al., 2016; Gorgoni et al., 2016; Winer et al., 2019).

### Tau Pathology and Local Sleep (T)

Habitual poor sleep and chronic sleep deprivation are associated with increased tau burden, tau hyperphosphorylation, and the spread of tau pathology across brainstem and hippocampal circuits, resulting in degeneration and memory impairment (Rothman et al., 2013; Di Meco et al., 2014; Ju et al., 2017; Zhu et al., 2018; Holth et al., 2019). Similar to the effects of sleep deprivation on Aβ, one recent finding indicated that ISF and CSF tau levels in rodents and humans also increase during wakefulness and decrease during sleep (Holth et al., 2019). Sleep deprivation exaggerated this wake-related increase, similar to its effects on Aβ, and chronic sleep deprivation increased the spread of tau pathology from the hippocampus to the locus coeruleus in the brainstem (Holth et al., 2019), a norepinephrine-expressing brain region known to accumulate tau pathology in the earliest stages of AD (Grudzien et al., 2007; Braak et al., 2011; Ehrenberg et al., 2017).

In addition to sleep deficits impacting tau pathology, more recent work shows that tau burden may be related to a number of distinct global and local sleep deficits observed in MCI and AD, each of which likely depends on the location of tau accumulation. Alongside the initial deposition of tau in the medial temporal lobe (MTL), tau also deposits in the brainstem, midbrain, and hypothalamus, preferentially within many sleep and wake-promoting nuclei (Oh et al., 2019), and this may decrease the consolidation of sleep and wakefulness which are regulated by these circuits (Lim et al., 2013a, 2014). Within the MTL, tau burden disrupts the GABA-regulated temporal synchrony of hippocampal ripple events during sleep and quiet wakefulness in a rodent model of tauopathy (Witton et al., 2016). Another rodent model of tauopathy identified the evolution of changes in sleep physiology, such that increases in NREM delta power and REM theta power in P301S tau transgenic mice relative to wild type mice at 6 and 9 months of age was followed by a decrease in NREM delta and REM theta power at 11 months (Holth et al., 2017). This finding indicated that, similar to the effect of MCI and AD on hippocampus activation, there may be a u-shaped curve in the expression of NREM and REM sleep oscillations as tau burden increases in the cortex.

In human studies, sleep spindle density and duration, but not SWA, are negatively associated with CSF-measured total and hyperphosphorylated tau levels in cognitively asymptomatic older adults (Kam et al., 2019). A similar finding was observed with slow wave-sleep spindle coupling strength, over frontal and parietal derivations, in relation to MTL tau burden in cognitively asymptomatic older adults (Figures 1B,D) (Winer et al., 2019). Similar to (Kam et al., 2019), this study did not find an association between SWA and MTL tau burden. In contrast, other reports showed that cortical tau was associated with a global loss of SWA across frequencies and prolonged duration of slow wave hyperpolarized down states (Menkes-Caspi et al., 2015; Lucey et al., 2019). However, these studies included patients with MCI (Lucey et al., 2019), and animal models that deposit tau in the cortex (Menkes-Caspi et al., 2015), which is not typical of early preclinical AD stages (Braak and Braak, 1996). It is possible that when tau remains confined to MTL and brainstem regions in early stages, SWA is not affected, while sleep features that depend on these structures are affected. Once tau begins to spread cortically, global deficits in SWA may begin to be apparent (Figures 1A,D), drowning out the hyperexcitability effects of Aβ (Busche et al., 2019). This hypothesis, which remains to be tested, would indicate that global and local sleep deficits due to AD pathophysiology are not static across disease stages, but instead evolve across disease stages as distinct AD pathophysiological features build up and spread throughout various cortical and subcortical brain circuits. If true, this would mean that a comprehensive picture of global and local sleep deficits may offer unique insight into how AD pathophysiology is progressing in a given individual, even in preclinical stages. Whether these tau effects on sleep are prior to or following tau-related neurodegeneration remain unclear, and should be distinguished in future studies.

### Neurodegeneration and Local Sleep (N)

Similar to tau pathological burden, the influence of neurodegeneration on global and local sleep likely depends on the location of neurodegeneration. Moreover, the distinction between degeneration due to age-related processes and dementia is not always clear. In the context of global sleep, loss of galanin-expressing inhibitory neurons in the intermediate nucleus of the hypothalamus, a potential human homolog to the ventral lateral preoptic nucleus which regulates sleep maintenance, is associated with sleep fragmentation (Lim et al., 2014). In patients with AD, the loss of galanin neurons and sleep fragmentation were both more extreme, but the overall relationship remained unchanged. Hence, it is not clear whether galanin neuron degeneration was due to an AD-specific process that began early in preclinical stages, or whether it was primarily age-related. However, there was also a trend for neurofibrillary tau tangles to be more concentrated in those with greater sleep fragmentation, indicating that galanin neurodegeneration may be driven by tau pathological burden in the hypothalamus (Lim et al., 2014). New evidence may help begin to distinguish between the role of tau and tau-related neurodegeneration on sleep expression. A recent study conducted comprehensive histopathological analysis of tau inclusion, neurotransmitter synthesis, and neuronal loss in a series of brainstem wake-promoting nuclei in healthy controls and patients with a variety of tauopathies, including AD, corticobasal degeneration (CBD), and progressive supranuclear palsy (PSP) (Oh et al., 2019). Increased tau inclusion and a decrease in neurons synthesizing neurotransmitters was apparent in all tauopathies relative to controls. However, it was only in AD that widespread neuronal loss was observed. Given these findings, it is therefore possible that distinct sleep phenotypes observed in distinct tauopathies is driven not by tau pathology, *per se*, but by the neurodegenerative effects of that pathology. For example, an extreme form of insomnia characterized by the inability to nap despite a profoundly shortened nighttime sleep duration (2–4 h per night) is consistently seen in PSP patients (Gagnon et al., 2008; Walsh et al., 2017), while sleep/wake instability is more typical in AD patients (Gagnon et al., 2008). This makes sense if widespread neuronal loss in wake-promoting brainstem regions is observed in AD and not in PSP. The reason why tau-related neurodegeneration in the brainstem differs by tauopathy remains unclear, though one possible mechanism regards potential differences in interactions between central inflammation and tau pathology (Leyns and Holtzman, 2017).

Less is clear about the role of neurodegenerative processes in the expression of local sleep. While it has been shown that frontal atrophy is consistently associated with slow wave deficits and slow wave-sleep spindle coupling deficits (Mander, 2013; Varga et al., 2016a; Helfrich et al., 2018), and hippocampal atrophy and frontal white matter degeneration are associated with sleep spindle deficits (Fogel et al., 2017; Mander et al., 2017b), it is unclear if these are secondary to an AD process or generally related to aging. However, recent findings have linked deficits in subjective sleep duration and quality to cross sectional and longitudinal volumetric differences in signature cortical amnestic MCI and AD regions as well as overall ventricular enlargement (Lo et al., 2014; Spira et al., 2016; Alperin et al., 2019), though the directionality of these relationships remains unclear.

In terms of loss of EEG desynchrony during REM sleep in MCI and AD, a double blind placebo controlled clinical trial administering donepezil, an anticholinesterase inhibitor, in patients with AD showed that donepezil treatment for 6 months reduced REM sleep EEG slowing and increased REM sleep duration (Dos Santos Moraes et al., 2006). This indicates that REM sleep EEG slowing may be due to progressive loss of cholinergic activity in AD (Figures 1C,D). This is consistent with the fact that EEG desynchrony during REM sleep is critically dependent on brainstem and basal forebrain cholinergic inputs (Peever and Fuller, 2016; Scammell et al., 2017), and that basal forebrain cholinergic projections are selectively degenerated early in the AD pathophysiological process (Mufson et al., 1988; Mesulam et al., 2004; Oh et al., 2019). These findings support the hypothesis that REM sleep EEG slowing may be related to tau pathological burden, but only because of the tau-dependent degeneration of the cholinergic system (Oh et al., 2019). However, these hypotheses remain to be directly tested. Further, whether global and local REM sleep deficits are a biomarker of or directly related to cognitive deficits in AD is unknown.

Hence, new emerging evidence indicates that signature pathological features of AD (i.e., Aβ, tau, and neurodegeneration) are all associated with distinct effects on global and local sleep expression, and that the sleep deficits observed in AD are likely dependent on the location and relative severity of each of these processes and the manner in which they synergistically interact. However, much less is known about the direct and indirect roles these local sleep deficits may play in the cognitive decline associated with AD pathophysiology.

## Local Sleep and AD-Related Memory Impairment

Critical components of a functioning MTL memory system include a network linking the entorhinal cortex (EC) layer II neurons to the dentate gyrus (DG) and cornu ammonis 3 (CA3) hippocampal subfields by way of the perforant path (Hyman et al., 1986; Witter, 2007). This network, which supports successful separation between similar yet distinct memories—i.e., pattern separation (Yassa et al., 2011b)—is highly sensitive to age and sleep loss, and is among the earliest and most dramatically affected in AD (Jagust, 2013; Leal and Yassa, 2013). Indeed, degeneration of this MTL circuit is a critical preclinical structural biomarker of MCI and AD (Desikan et al., 2012; Holland et al., 2012). Targeted studies have identified diminished pattern separation ability both in aging (Wilson et al., 2006; Stark et al., 2010, 2013; Yassa et al., 2011a,b) and following sleep deprivation (Saletin et al., 2016), and impaired pattern separation is even more dramatic in patients with amnestic MCI (Bakker et al., 2012, 2015). In aging and MCI, these deficits are associated with DG/CA3 region hyperactivity (Yassa et al., 2011a,b), EC thinning (Reagh et al., 2018), and perforant path degeneration (Yassa et al., 2010a, 2011b). Levetiracetam (LEV) is an anti-epileptic drug that modulates synaptic excitability by binding to synaptic vesicle protein SV2A, thus impacting action potential-dependent neurotransmitter release (Lynch et al., 2004). LEV administration in aMCI patients significantly reduces hyperactivity and enhances object pattern separation performance relative to placebo (Bakker et al., 2012, 2015), supporting the notion that this hyperactivity is dysfunctional rather than compensatory. Of note, in addition to its impact on functional hyperactivity and cognition during wakefulness, LEV has also been shown to consolidate SWS and increase its duration in healthy adults, suggesting that it may also influence SWS-related memory processing as well (Cicolin et al., 2006).

Beyond the MTL, cortical network dysfunction and impaired functional connectivity have been observed in the context of AD pathophysiology. In particular, breakdown of connectivity and disinhibition of activity within the default mode network (DMN) is associated with both hippocampus hyperactivity and cognitive impairment in individuals with AD pathologies (Pihlajamaki et al., 2008; Jacobs et al., 2013; Pereira et al., 2019). Disruptions in DMN functional connectivity are also observed following sleep deprivation (Gujar et al., 2010) and are associated with the extent of daytime sleepiness (Ward et al., 2013). This may be due, in part, to a deprivation or disruption of infraslow EEG oscillations during SWS that organize DMN expression supporting sleep-dependent memory consolidation (Picchioni et al., 2011). Together, these findings implicate widespread circuit dysfunction within frontal, parietal, and MTL networks, which are associated with memory impairments even prior to AD-related neurodegeneration. Further, dysfunction within this circuit has also been associated with sleep disturbance, including in disorders such as sleep apnea and attention deficit hyperactivity disorder (Gujar et al., 2010; Ward et al., 2013; Khazaie et al., 2017; Tashjian et al., 2017), indicating that sleep disturbance may also be related to the effects of AD pathophysiology on cortical brain function.

The role of AD pathology in this memory-related circuit dysfunction is still being investigated. However, it appears that MTL tau burden, microglial dysfunction, and related synaptic loss are more strongly associated with memory impairment than Aβ burden (Jack et al., 2010, 2013; Scholl et al., 2016; Spangenberg et al., 2016; Pereira et al., 2019). This has led to the view that tau pathology is more directly linked with cognitive impairments in AD than Aβ, though the truth is likely more complicated (Bloom, 2014; Maass et al., 2018; Sperling et al., 2019). For example, while tau is more likely to deposit early in the MTL, the tau-related memory impairments observed may be more typical of age-related memory decline (Maass et al., 2018). Once tau spreads beyond the MTL, which may depend on the presence of Aβ (Bloom, 2014; Tosun et al., 2017; Sperling et al., 2019), AD-related cognitive decline may begin to emerge and progress (Tosun et al., 2017; Sperling et al., 2019). Regardless, the pathophysiology of the long-term trend in tau-related cognitive decline may be distinct from the memory impairments due to hippocampus hyperexcitability.

What role, if any, that local sleep dysfunction plays in AD-related memory impairment remains unclear, though there are multiple theoretical ways in which local sleep could interact with AD pathologies to disrupt a variety of memory functions. The state of the science supporting each of these possibilities is reviewed below.

### Direct Effects

An established literature demonstrates a clear relationship between local sleep features and multiple forms of memory encoding and consolidation (Fischer et al., 2002; Walker et al., 2002; Marshall et al., 2004, 2006; Spencer et al., 2006, 2007; Mander et al., 2011, 2013, 2014, 2015, 2017a,b; Antonenko et al., 2013; Fogel et al., 2013, 2017; Westerberg et al., 2015; Lustenberger et al., 2016; Papalambros et al., 2017). Three, non-mutually exclusive core theories of sleep-dependent memory consolidation implicate specific synaptic and systemic processes that mechanistically support the role of sleep in the long-term retention of procedural and episodic memories. There is evidence that many of the neurobiological mechanisms supporting these process may be directly disrupted by AD pathophysiology.

**FIGURE 3:**
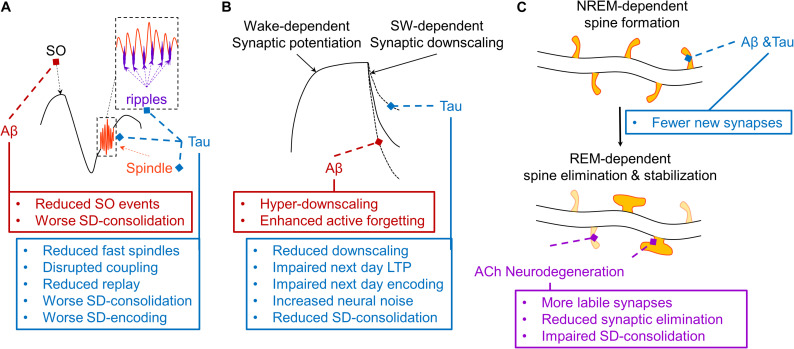
Schematic depiction of hypothesized effects of AD pathophysiology on mechanisms of three models of sleep-dependent memory consolidation. **(A)** A theoretical depiction of the mechanisms supporting active systems consolidation (Steriade, 2006; Diekelmann and Born, 2010; Watson and Buzsaki, 2015). The thalamic sleep spindle (orange) is phase-locked to the depolarizing upstate of the slow oscillation (SO, black), and the hippocampal ripple is phase-locked to the troughs of the sleep spindle (purple), supporting coordinated replay of memory traces which triggers the cortical plasticity necessary for systems consolidation. Aβ (red) selectively disrupts SO expression, impairing sleep-dependent (SD) memory consolidation. Tau (blue) disrupts fast sleep spindle and ripple expression and their coupling with the SO, disrupting memory replay and related memory consolidation and subsequent encoding (Walker, 2009; Watson and Buzsaki, 2015; Mander et al., 2017a). **(B)** A theoretical depiction of the sleep homeostasis hypothesis (SHY), which posits that slow waves (SW) facilitate global synaptic downscaling in response to widespread synaptic potentiation following waking experience (Tononi and Cirelli, 2014). Aβ increases delta waves, which may increase the magnitude of synaptic downscaling, potentially overriding local signals protecting relevant synapses from long-term depression (LTD), ultimately exaggerating an active forgetting process. Tau reduces overall slow wave activity, which may weaken the process of synaptic downscaling, resulting in increased neural noise and impaired sleep-dependent memory consolidation. Another consequence could be a brain that remains more over-potentiated, thus limiting the capacity to induce long-term potentiation (LTP), resulting in impaired next day learning. **(C)** A theoretical depiction of a NREM-REM two-stage model of synaptic plasticity supporting sleep-dependent memory (Yang et al., 2014; Miyawaki and Diba, 2016; Li et al., 2017; Mizuseki and Miyawaki, 2017). In this model, NREM sleep facilitates the formation of a small number of dendritic spines on dendritic branches of neurons triggered by learning experiences during prior wakefulness. This is followed by a REM sleep-dependent process that eliminates most of these new labile dendritic spines, but stabilizes some that most support successful memory formation. Through their disruption of memory-relevant NREM sleep oscillations, both Aβ and tau could reduce the number of new dendritic spines formed following a learning experience. Through cholinergic degeneration (purple), and the resulting impairments in REM sleep physiology, this could be further compounded by impaired REM sleep-dependent elimination and stabilization of relevant dendritic spines. This could result in greater neural noise, faster memory trace decay, and a reduction in resistance to interference, exacerbating forgetting.

First, one theory posits that the coordinated expression of cortical slow oscillations (0.5–1 Hz), cortico-thalamic sleep spindles (12–16 Hz), and hippocampal ripples (140–220 Hz) actively supports memory consolidation by triggering memory replay during SWS to transform memory traces to be more cortically-dependent (Figure 3A) (Steriade, 2006; Diekelmann and Born, 2010; Abel et al., 2013; Staresina et al., 2015; Mander et al., 2017a; Helfrich et al., 2018). This framework has been supported by studies demonstrating induction of LTP-related plasticity, coordinated replay of neuronal ensembles, and coordinated hippocampal-neocortical information transfer during this tripartite coupling of slow wave, sleep spindle, and ripple events (Rosanova and Ulrich, 2005; Wierzynski et al., 2009; Chauvette et al., 2012; Abel et al., 2013; Latchoumane et al., 2017; Helfrich et al., 2019). More specifically, it has been shown that the hyperpolarized downstate of the cortical slow oscillation induces the expression of sleep spindles in the thalamic reticular nucleus which are preferentially expressed during the rising phase toward the depolarizing upstate of the slow oscillation (Mak-McCully et al., 2017). In addition, hippocampal ripple expression is maximal during the excitatory troughs of sleep spindles (Staresina et al., 2015; Latchoumane et al., 2017). Recent closed-loop optogenetic evidence in rodents has demonstrated that this precise phase-relationship of coupling between sleep spindles with slow oscillations is essential for successful hippocampus-dependent memory consolidation (Latchoumane et al., 2017). Induction of sleep spindle expression in thalamic reticular neurons during the rising phase of the slow oscillation up-state increases phase locking of hippocampal ripples to spindle troughs and enhances memory consolidation, while suppression of sleep spindle expression during this SO phase impairs memory consolidation. Conversely, either inducing or suppressing sleep spindle expression outside of this SO rising up phase has no effect on memory consolidation. Prior studies offer mechanistic support for this effect of SO-sleep spindle coupling on memory consolidation. Sleep spindles have a local effect on neuroplasticity, but this depends on its coupling with slow oscillations. When sleep spindles are appropriately phase locked to slow oscillations local evidence of long term potentiation (LTP) is apparent, and when sleep spindles are expressed without slow oscillations long term depression (LTD) is instead observed (Rosanova and Ulrich, 2005). Another recent optogenetic study also demonstrated a causal role for SO events (<1 Hz slow waves) and not faster frequency delta waves (1–4 Hz slow waves) in memory retention (Kim et al., 2019). Hence, optogenetic manipulation of both SO and sleep spindle events directly impacts long-term memory retention, supporting their causal role in sleep-dependent memory consolidation. This model is further supported by studies that mechanistically enhance slow waves, sleep spindles, and slow wave-sleep spindle coupling through external electrical and auditory stimulation methods in humans, resulting in improved procedural and episodic memory, even in older adults and patients with MCI (Marshall et al., 2004, 2006; Westerberg et al., 2015; Lustenberger et al., 2016; Ladenbauer et al., 2017; Papalambros et al., 2017, 2019).

Theoretical depictions of this form of sleep-dependent memory describe a process by which hippocampal-neocortical neuronal ensembles which are primed by learning during prior wakefulness are reactivated during slow wave sleep in a temporally precise manner. This is supported by classic studies showing replay of coordinated place cell activity during slow wave sleep, and specifically during hippocampal ripples (Wilson and McNaughton, 1994; Ji and Wilson, 2007; Davidson et al., 2009). It has been hypothesized that this replay provides a window for information transfer from hippocampal nodes to neocortical nodes facilitating neuroplasticity in support of memory transformation and long-term memory retention (Buzsaki, 1998). This supposition has been supported by studies in rodents and intracranial EEG studies in humans describing evidence of information transfer from hippocampus to cortex during triple coupling of SOs, sleep spindles, and ripple events (Olcese et al., 2018; Helfrich et al., 2019), with this information transfer being particularly prominent in neuronal ensembles modulated by a memory task in prior waking periods (Olcese et al., 2018). Further support for the causal role of replay in memory consolidation is demonstrated by a series of studies implementing targeted memory reactivation (TMR) methods to trigger the replay of memories encoded prior to sleep during SWS. This approach implements pairing learning with external sensory stimulation, such as pairing odor cues with a memory task (Rasch et al., 2007) and auditory cues with individual memory trials (Rudoy et al., 2009; Antony et al., 2012; Van Dongen et al., 2012; Batterink et al., 2016; Berkers et al., 2018). These sensory cues are then replayed during sleep with memory performance being assessed in sensory cued versus un-cued trials in subsequent waking periods. These studies have shown that sensory cueing specifically during SWS enhances slow wave-sleep spindle coupling and memory performance (Rasch et al., 2007; Rudoy et al., 2009; Antony et al., 2012; Van Dongen et al., 2012; Feld and Diekelmann, 2015; Batterink et al., 2016; Berkers et al., 2018; Bar et al., 2020), with the efficacy of this effect depending on the phase of the slow wave during sensory cueing (Batterink et al., 2016; Goldi et al., 2019). Hence, these findings implicate a mechanism by which triple coupling of SO, sleep spindle, and ripple events coordinate replay of relevant neuronal ensembles supporting targeted neuroplasticity in facilitation of memory consolidation.

Cortical slow oscillations are diminished in the presence of both Aβ and tau pathology in the cortex (Mander et al., 2015; Lucey et al., 2019), and this effect is independent of gray matter atrophy (Mander et al., 2015). Furthermore, this effect of Aβ on SWA was correlated with retrieval-related hippocampus hyperactivation, which was negatively associated with sleep-dependent memory retention (Mander et al., 2015). Further supporting the importance of the <1 Hz SO, doubling the frequency of the SO from 0.6 to 1.2 Hz in APP transgenic mice resulted in reduced dendritic spine density, likely directly impacting memory functions (Kastanenka et al., 2019). In addition, MTL tau burden has been shown to disrupt hippocampal ripple expression and slow wave-sleep spindle coupling during NREM sleep (Witton et al., 2016; Winer et al., 2019). Hence, current evidence indicates that AD pathologies disrupt the expression and coupling of all three of the core sleep oscillations underlying sleep-dependent memory consolidation (Figure 3A). Beyond their direct effects on local sleep expression, AD pathophysiology could also disrupt cortical network structure and function in a way that diminishes the efficacy of local sleep expression to support consolidation across memory systems, as observed in aging (Mander et al., 2017a).

Second, the synaptic homeostasis hypothesis posits that slow waves act to globally downscale synapses to manage the growing energy demands of the brain following continuous learning and thus wide-spread synaptic potentiation (Figure 3B) (Tononi and Cirelli, 2014). In theory, memory is consolidated and enhanced by this process as it acts to enhance the signal to noise of neuronal ensembles related to a given memory trace. A series of studies across model systems support key tenants of this hypothesis. Synaptic potentiation is saturated following extended wakefulness, resulting in partial occlusion of long term potentiation (LTP) induction, which is refreshed following SWS (Vyazovskiy et al., 2011). This is mirrored in some human studies, with slow wave stimulation and sleep spindle expression both predicting restoration of memory encoding ability following sleep (Mander et al., 2011; Antonenko et al., 2013). Other studies have shown that memory tasks targeting localized brain networks result in local increases in SWA in subsequent sleep periods, with the intensity of SWA predicting the degree of motor learning (Huber et al., 2004, 2006). A recent optogenetic paper has showed that while SO events support memory retention, delta waves may support forgetting (Kim et al., 2019). This finding may support the presence of both hypothesized mechanisms in SWS, with an active process enhancing synaptic strength of a small number of neuronal ensembles directly relevant to triggered replay of specific prior waking experiences, while another globally downscales synapses to maintain energy homeostasis and reduce neural noise.

This latter mechanism may also be influenced by AD pathology (Figure 3B). While Aβ decreased SO events, it also increased delta waves (Mander et al., 2015). It is possible this shift in SO-delta wave balance may shift memory systems toward greater forgetting and lower memory retention (Kim et al., 2019). Indeed, while SO events were positively associated with sleep-dependent memory consolidation, delta events were negatively associated with sleep-dependent memory consolidation in older adults with Aβ pathology (Mander et al., 2015). In addition to this potential “hyper downscaling” in the presence of Aβ, cortical tau may impoverish the downscaling process altogether, indirectly impacting the ability of the brain to encode novel experiences in subsequent waking periods due to incomplete depotentiation of synapses (Figure 3B). Therefore, AD pathologies may either exaggerate a downscaling process or disrupt it altogether, impairing sleep-dependent memory consolidation and/or sleep-related memory encoding ability.

Lastly, another model concerns coordinated sequential synaptic remodeling across NREM-REM cycles (Yang et al., 2014; Miyawaki and Diba, 2016; Li et al., 2017; Mizuseki and Miyawaki, 2017). This theory is supported by animal work showing that NREM sleep can trigger a limited formation of new synapses following a learning experience in a dendritic branch-specific manner (Yang et al., 2014). REM sleep then eliminates many of these, but stabilizes the synapses not eliminated (Li et al., 2017). AD could impact this process, too, by disrupting the stability of NREM and REM sleep, shortening REM duration, and/or disrupting the EEG desynchrony characteristic of REM sleep. This would likely result in fewer new synapses formed following learning, all of which would be more labile and vulnerable to decay (Figure 3C). As with the other sleep-dependent memory models, this possibility has not been directly tested.

Each of the theoretical mechanisms proposed to support sleep-dependent memory encoding and consolidation are likely negatively influenced by AD pathophysiology (Figure 3), which would contribute to the memory impairments observed in AD. However, the impact of local sleep deficits on memory in AD is not necessarily limited to the effects of AD pathophysiology on sleep-dependent memory mechanisms.

### Indirect Effects

There is also evidence that AD pathology interacts with sleep deficits to influence cognition. For example, the interaction between Aβ and sleep efficiency predicts cognitive deficits on cognitive tasks, such that poor sleep quality results in impaired memory to a greater extent when an individual has AD pathology in their brain (Molano et al., 2017). Moreover, the influence of APOE genotype—the strongest genetic risk factor for late onset, sporadic AD (O’Donoghue et al., 2018)—on cognition depended on the quality of sleep, with APOE genotype having minimal impact in those with high quality sleep and substantial impact on those with low quality sleep (Lim et al., 2013b). This indicates that sleep may be related to a neural reserve factor making the brain more or less vulnerable to the presence of AD pathology or risk factors. Indeed, there is a literature that indicates that deficits in sleep quantity and quality result in cortical brain atrophy (Spira et al., 2016; Alperin et al., 2019) and overall ventricular enlargement even over the course of 2 years in healthy older adults (Lo et al., 2014), which would likely diminish neural reserves to compensate for AD pathophysiology.

Another possibility is that sleep may be related to cognitive impairment independently of sleep-dependent memory processes through its direct effects on AD pathophysiology. Growing evidence indicates the bidirectional relationship between slow waves and Aβ and tau pathology (Zhang et al., 2005; Xie et al., 2013; Mander et al., 2015, 2016; Menkes-Caspi et al., 2015; Varga et al., 2016b; Ju et al., 2017; Kastanenka et al., 2017, 2019; Kent et al., 2018; Fultz et al., 2019; Lucey et al., 2019). More specifically, slow wave deficits and sleep/wake instability appear to foster greater production and lower clearance of both Aβ and tau pathology (Zhang et al., 2005; Mander et al., 2015; Varga et al., 2016b; Ju et al., 2017; Kastanenka et al., 2017, 2019; Kent et al., 2018; Musiek et al., 2018; Lucey et al., 2019), which may then exacerbate central inflammation and MTL-related neurodegeneration, thus accelerating memory decline (Jack et al., 2010, 2013, 2019; Yassa et al., 2010b, 2011b; Desikan et al., 2012; Holland et al., 2012; Jagust, 2013; Leal and Yassa, 2013; Scholl et al., 2016; Spangenberg et al., 2016; Leal et al., 2017; Spangenberg and Green, 2017; Reagh et al., 2018; Pereira et al., 2019).

Hence, in addition to the disruption of local sleep processes related to sleep-dependent memory encoding and consolidation, sleep disruption may further accelerate AD pathophysiology or make the brain more vulnerable to it. Each of these could contribute to AD-related cognitive decline, and could do so at distinct stages. For example, sleep-dependent memory deficits may emerge as soon as local sleep deficits emerge in the initial stages of AD pathogenesis (Mander et al., 2015), while deficits associated with neural reserve or AD-related neurodegeneration may emerge later once AD pathological burden has reached a tipping point or degeneration has progressed sufficiently to result in clinical symptoms (Westerberg et al., 2012; Lim et al., 2013b; Liguori et al., 2014). This would indicate that the role of sleep in AD-related cognitive decline may also be multifaceted and evolve across AD pathophysiological stages.

## Conclusion and Future Directions

Evidence continues to mount in support of the relationship between local sleep processes and AD pathophysiology. Recent studies have established that Aβ, tau, and neurodegeneration, defining pathological components of AD, all result in specific deficits in local and global sleep expression that depend on the underlying brain regions impacted (Figure 1). Pathological accumulation and degeneration within brainstem and hypothalamic sleep/wake regulatory centers fosters sleep state instability, pathology and degeneration in the MTL impacts ripple and spindle expression, cortical deposition and degeneration alters slow wave expression, and AD-related cholinergic degeneration likely disrupts REM sleep expression. These deficits are likely not static, but evolve as AD pathophysiology progresses through brain networks and across disease stages. For example, the slow wave deficits due to Aβ may differ from those observed with tau (Figure 1A). In some cases, these deficits may actively contribute to the progression of the disease, especially as they relate to the facilitation of Aβ and tau accumulation and neurodegeneration of circuits vulnerable to these pathologies (e.g., disruption of glymphatic clearance of AD pathologies). There is some evidence that these associations between AD pathophysiology and local sleep expression may be relevant for the memory impairments observed in the context of AD, though mechanisms of these relationships remain hypothetical.

The current state of the science indicates that there are more questions that remain than answers, both at mechanistic and therapeutic levels. At the mechanistic level, a clearer understanding of how local sleep is influenced by AD pathophysiology at each stage of the disease process is warranted. This means conducting larger scale studies that can implement the ATN framework to understand how the presence and absence and relative burdens of different AD pathologies at different AD stages may impact local sleep. This will also require examining other mechanisms, including metabolic dysfunction, chronic inflammation, and HPA-axis dysregulation, all of which contribute to AD pathophysiology and are related to sleep disturbance (Heppner et al., 2015; Spangenberg and Green, 2017; Caruso et al., 2018; Carroll and Macauley, 2019; Irwin and Vitiello, 2019; Chappel-Farley et al., 2020). Chronic inflammation, in particular, is a promising target for future research, because of its known independent interactions with sleep expression and multiple aspects of AD pathophysiology, as well as the paucity of data addressing its links with age-related deficits in local sleep expression. Further, targeted studies examining how the flow of CSF in the brain that is phase-locked to SWA (Fultz et al., 2019) may change with age and in relation to endogenously generated pathological features of AD will critically advance our understanding of the importance of the glymphatic system in AD pathogenesis. A greater understanding of the intersection between sleep disorders and local sleep, and their relationship with AD pathophysiological progression is needed, to better understand how various forms of sleep disturbance impact AD risk. Studies examining how local sleep differs across forms of dementia or kinds of AD will be informative both in terms of understanding mechanisms and the utility of local sleep to aid differential diagnosis. Longitudinal studies incorporating local sleep measures alongside AD biomarkers are of great need to determine the time course of sleep changes in relation to AD pathophysiology. Lastly, a comprehensive examination of how local sleep disruption is related to various forms of impairment in sleep-dependent and sleep-independent cognitive processes in relation to AD pathophysiology will help unpack the role(s) sleep plays in AD-related cognitive decline across disease severity. This will necessarily include examination of the influence of MCI, AD, and AD pathophysiology on ripple expression and memory replay, as well as the efficacy of targeted memory reactivation paradigms to facilitate memory transformation and consolidation.

Alongside these efforts, an emphasis on large-scale studies implementing sleep interventions must be considered. At present, it is not clear whether local sleep deficits are a biomarker of AD progression, or are active contributors to AD pathophysiology or related cognitive decline. The utility of sleep interventions, both targeting global sleep and local sleep, to address AD pathophysiological progression remains entirely unknown. While there are some data that suggest that treating sleep disorders may impact AD biomarkers (Osorio et al., 2015; Liguori et al., 2017a,b), large scale studies are lacking. Further, while a few reports show that enhancing local sleep in older adults and patients with MCI will improve overnight memory consolidation (Ladenbauer et al., 2017; Papalambros et al., 2019), the efficacy of this type of intervention, even for more than one night, is unknown. As important is an examination of the impact of AD disease stage on the efficacy of sleep interventions to impact AD pathophysiology and related cognitive impairments. Will sleep interventions help preclinical, MCI, or AD patients equally, or will sleep interventions have diminishing returns as the disease progresses, or affect distinct outcomes? This will be a critical question to answer, as it will help guide expectations related to disease management and identify the appropriate therapeutic targets based on the disease stage.

While much remains unknown, it is clear that local sleep processes are intricately intertwined with AD pathophysiology, likely in a bidirectional manner and through multiple mechanisms. These findings also open up the possibility that local sleep may offer a unique and sensitive window into circuit level dysfunction across different forms of dementia, aiding early efforts in differential diagnosis of dementia risk. As different forms of dementia target distinct neural networks, the profile of deficits in local and global sleep appear to differ as well (Petit et al., 2004; Gagnon et al., 2008; Oh et al., 2019). Ultimately, the importance of local sleep for the optimal functioning of the brain means that local sleep disruption will likely be a crucial factor in the progression of all dementias, whether as a biomarker of a diseased brain, a contributor to disease pathogenesis, or an independent factor influencing the brain’s resiliency or vulnerability to dementia. Uncovering the specific roles of local sleep in dementias such as AD will thus offer new opportunities to target interventions to offer symptomatic relief, impact disease progression and risk, and slow affiliated cognitive decline.

## Author Contributions

The author confirms being the sole contributor of this work and has approved it for publication.

## Conflict of Interest

The author declares that the research was conducted in the absence of any commercial or financial relationships that could be construed as a potential conflict of interest.
